# Healing of Gun-Shot Wounds by First Intention

**Published:** 1864-07

**Authors:** Middleton Michel

**Affiliations:** Surgeon P. A. C. S.


ART. II.—*Healing of Gun-Shot Wounds by First Intention.* By MIDDLETON MICHEL, Surgeon P. A. C. S.

The past three years' experience of the ravages of the minie ball in its destructive ploughing up of the textures while inflicting its characteristic wound, would scarcely appear to indulge the inquiry whether such lesion can ever possibly heal by first intention.

The contingent circumstances which could permit the transit of the conical ball, in such a manner, through the several planes of the different surfaces and fibres, of all the structures, as simply to separate or divide, rather than contuse or lacerate them, would imply a series of coincidences so remarkable in themselves, as would appear to render such an occurrence wholly improbable. The very announcement that a gun-shot wound, which we know must suppurate, should fall into appositions throughout its continuity, pour out plastic lymph spontaneously, assuming organization, and re-establish union, appears to subvert the best ascertained and most reliable dogmas in the science of surgery; yet, we are prepared to state that, notwithstanding the difficulties of the question and the insufficiency of many attempts at a reasonable explanation, such a wound may present the phenomenon of spontaneous cure: that is, without suppurating, at most exhaling only from its surface that amount of formative product interposed to bring the surfaces into more speedy coherence.

That small size round-shot, or even the round ball, should penetrate a part, dissect up the textures, and produce no greater disturbance than is looked for from the puncture of a sharp instrument, is a matter of no great wonder. All the old writers, Hunter among them, furnish occasional instances of immediate re-union in superficial injuries by the round ball. More modern authorities, such as Larrey and Sanson, Vidal de Cassis, and others, have absolutely seen the joints transfixed, and though clearly opened, the case progress with rapidity and little inconvenience to a perfect cure; but, that the conical ball should deport itself sometimes in the same way, we are ready to confess we were not willing to admit, until a broad reference to the Surgeons of the Confederate Army supplied us with results of an experiance too significant to omit mention in detail.

Surgeon A. M. Fauntleroy, Medical Director of the department of North Carolina, has communicated to us the case of a private of the 8th Louisiana regiment, who was shot by the accidental discharge of a companion's gun, (rifle-minie.) The ball entered the inside of the left foot, coursing the arch of the instep, coming out at a point opposite its entrance. Suppuration never occurred, and the wound healed by first intention. At the battle of Drainsville, Surgeon E. S. Gaillard had under his notice a private whose gastrocnemius muscle was perforated by a minie ball, yet this party recovered without suppuration. Surgeon W. S. Mitchell, Chief Surgeon of Rodes' division, informs me, that Lieutenant E., of the 12th Georgia regiment, was wounded by a minie ball, which entered about half an inch above the dorsum of the penis, to the right of the middle line, making its exit on the outer and posterior portion of the right buttock. There was no suppuration. The lieutenant was never confined to bed, and the orifice of exit was entirely closed by the third day. The orifice of entrance closed almost immediately by scab, which remained six or eight days.

Surgeon J. B. Read gives me the case of Lieutenant N. R., of the 10th Virginia cavalry, wounded June 20th, 1863, at the cavalry battle of Brandy Station. A minie ball entered the sixth intercostal space, on right side, making its exit at fourth intercostal space of the same side, three-quarters of an inch externally to the nipple. Expectoration of blood and escape of some bubbles of air were observed, but all of this disappeared by the 30th June. No suppuration occurred, and at this period, after the injury, no scabs existed on either wound. Auscultation revealed a perfectly healthy lung.

There seems to be some common features of resemblance between the instances above recorded, deserving of notice, as perhaps indicating the conditions most favorable towards such results. These are—the superficial nature of the injuries, the obliquity, and, perhaps, valvular character of the wounds, and the short range of fire, at least in two of the of cases, the ball entering very soon after its discharge from the piece, with its initial velocity, which we know is the maximum velocity of a ball. In one instance, the missile follows the convexity of the instep, in the second case, notwithstanding the diversity of sentiment as to its passage through the abdomen, there is little doubt of its having coursed along the walls of that cavity, and in the chest wound, as sometimes happens, the ball passed, in all probability, between the lung and the walls of the thorax without seriously wounding the pleura, and certainly not the lung, since careful auscultation disclosed not the slightest impairment in the functions of that organ. Nor will it be argued that the expectoration of blood, or even escape of air, are indications to the contrary, since these phenomena are not unequivocal evidences of lesion of this organ.

But what is far more remarkable in this connection, we have still to record an equally favorable termination, where more serious injury has been inflicted, involving deeper seated organs, and complicated with fracture. Assistant-Surgeon McQueen, of Daniel's brigade, reports that after the battle of Chancellorsville, among the wounded remaining in his charge at the "Lacey" house, was a man who presented a compound comminuted fracture of the upper-third of the femur, from a minie ball, and the wound healed in less than one week, without suppuration. Surgeon H. F. Campbell furnishes me the history of Lieutenant-Colonel ——, who was wounded through both thighs. The left thigh was fractured; the right presented a simple flesh wound. While the former underwent the reparation incident upon such an accident, suppurating abundantly, the flesh wound, though a deep one, healed promptly without any discharge, the patient himself manifesting some concern at the circumstance, under the impression, very common among the uninitiated, that his wound could not be doing well, since it did not discharge pus like the other.

The following cases, though open to the objection of not having been under the continued supervision of the surgeons who relate them from the commencement, are deeply interesting as exhibiting those powers, founded in the autocracy of nature, even in the presence of the gravest accidents of battle. Surgeon Read reports that Lieut. S——, of the 3d Georgia regiment, was wounded July 23d, 1863, at Manassas Gap, by a minie ball, which traversed both thighs just above the knees, lodging on the outer side of the right knee. The patient was in the act of stepping forward at the time. He lay four weeks without surgical treatment, with one thigh fractured; and, after sixty miles transportion in a wagon, reached the railroad which brought him to Richmond. On entering the hospital, under the able care of Surgeon Read, his wounds are found covered by dry scabs, and being a physician himself, he gives the assurance that there had been no suppuration. At the expiration of four weeks he had used crutches. There was consolidation, with one and a quarter inches of shortening on right side.

We are also indebted to Surgeon C. J. Clarke, of the Alabama Hospital, for the next: Jonathan Sykes, of the 13th Alabama regiment, at the battle of Chancellorsville, May 3d, 1863, was wounded through upper-third of the thigh, ball entering six inches below the anterior-superior spinous process of the ilium. He entered Dr. Clarke's hospital the 23d May. Neither the ball nor any fragments of bone had been removed; there had been no purulent discharge from the wound. The wound, at the time of his arrival, was completely healed; his limb was, however, placed on a double-inclined plane, with suspensory slings; no suppuration occurred during his stay in the hospital. He made a good recovery, with a limb shortened two inches.

The above cases, supported by all the evidence necessary fully to substantiate them, and their possible importance in a medico-legal aspect, will authorize a particular review of the principles of pathology, upon which they most likely depend.

The great danger dependent upon a compound comminuted fracture, accompanied by all the commotion or destruction of the soft parts which may be conceived to exist, is inflammation and its products; and experience teaches that the introduction of air into such a wound is, above all other causes, that most fraught with pernicious consequences; for it is such an accident that inevitably gives rise to high inflammatory action, and this, in its turn, to the production of pus, which interrupts, suspends, and inevitably postpones re-union. A more or less prompt healing by first intention under similar breaches of continuity, when the superjacent integuments are uninjured, is of daily occurrence; and a comminuted fracture, with loss of substance and such displacement of its payments as to produce considerable shortening of a limb, with laceration of the soft parts, injury of the periosteum, destruction of the muscles, their sheaths and surrounding cellular tissue, will often become repaired without the development of suppuration, if the introduction of air has been prevented. The extravasation of blood and exudants of a viscid and gelatinous consistency, are very soon transformed into the provisional and definitive callus and callulo-fibroid structures, which bridge the entire track of the injury, without evolution of the morbid products of inflammation. Therefore, it may be possible in certain exceptional and very rare conditions of a gun shot wound, in which the orifices, from their obliquity or valvular state, perfectly exclude the ingress of air, that it should find itself placed in no dissimilar condition from an ordinary comminuted fracture. The mere possibility of such an event is certainly exhibited in the illustrations above noted, while the extreme rarity of the occurrence is equally shown by the very few cases we have been able to obtain, from an army scattered over so large a part of the Confederacy, and after all the many hostile encounters in which it has been engaged.

From the relations in which we have sometimes found the walls of the bullet track in certain portions of their extent—that of nearly complete apposition, and in a short time of partial coherence—we must refer these examples of immediate adhesion to accidental coincidences in the special direction taken by the missile through the organs of the body, consorted, fortunately with its maximum velocity. To these physical conditions, so naturally and obviously interdicting the entrance of air, rather than to any peculiar constitutional healthiness on the part of the subject, would I refer so unexpected and favorable an issue. With these cases before us, then, we must conclude that, long as the contrary opinion has been entertained, it is now beyond doubt, that even deep-seated lesions of the character we have been examining, do occasionally heal by first intention.

Connate to this question, is the similar inquiry, whether this kind of union be more common where the injury affects the appearance of an incised wound.

That gun-shot wounds may assume such an aspect, when caused by the round ball, was known to Guthrie; and I have, myself, recently corroborated the statements of others, that the same effects may take place when the minie ball is the wounding agent.

M. Derigan, attached to the naval brigade before Sebastopol, noted the regularity of the edges of some of these wounds, and mentions instances in which the minie ball caused as clean a wound as if done by a sharp knife; the nose, in one instance, having been divided at the junction of the cartilages with the bones; the lower portion dropping down, but adhering by a good pedicle, and healing when brought together as in hare-lip. While recently among the wounded on the battle-fields of Spotsylvania and the Wilderness, I met three examples of perfect incised wounds produced by the conical ball, possibly at short-range, as they occurred during the attack upon our breastworks. All of these were about the head and face. Pieces of shell, fragments of foreign bodies, such as solid parts of the soldier's equipments, and bone itself from the fractured limb of a fellow-comrade, have been found to inflict wounds closely resembling incisions, but the minie ball also, as we have observed, may occasion such results when unchanged in shape during its flight, by striking at a considerable angle of incidence upon a broad and flat bone immediately subcutaneous, or over which the superjacent integument is tensely drawn; or where, again, it reaches some reduplication or fold of very laxed skin, such as obtains in the neighborhood of joints on the surface of either flexion or of extension, when the ball forces the integument into a cul-de-sac, producing a rent which, owing to the elasticity of the texture, appears even smaller than the object which has caused it. Such linear wounds have been far more frequent about the head than elsewhere, and exist as such only by conditional location, for we have repeatedly remarked in civil practice, similar effects under the agency of varied forms of wounding bodies when occurring about the scalp.

It has been questioned whether the edges of such wounds, more or less contused as they are found to be on nearer inspection, are any more suitable for primary adhesion. They unite, it is presumed, at least in part of their extent when brought in regular contact, and this is all that the experience of many teaches; but, from what I have seen of these wounds, I am disposed to hope for more frequent success when they are properly adjusted and in perfect apposition. From the worse looking lacerated wounds I have sometimes obtained excellent results, and in one of the three cases stated as occurring on the battle-field, the wound healed throughout its entire extent by first intention. This was a private of company "G," 3d South Carolina regiment, wounded through the face. One of the wounds presented so regular an incision through cheek and lip, that Surgeon J. Evans, who kindly exhibited it to me, brought down the flap and united its edges with sutures, and adhesion supervened promptly and so perfectly, that a linear cicatrix will perhaps be all that will hereafter indicate the accident.

The rapid closure of such wounds and exclusion of air when practicable, secures this mode of healing even in gun-shot wounds. Great stress should, therefore, be laid upon whatever means are within our reach in injuries about the head and face. Where the integument is loosely adherent and we can operate by *glissement*, the edges may be scarified, if not found in the accidentally incised state alluded to, and be brought together with sutures.

That such prompt cicatrization occurs after punctured wounds which depends doubtless upon the rapidity with which the track closes, through the elasticity of the separated tissues, I had several opportunities of ascertaining during the memorable fights of the 11th and 12th of May. In that remarkable assault on our breastworks, ten lines deep, in which the enemy exhibited unwonted boldness, and a persistent constancy of purpose only interrupted by night and only terminated by a disastrous repulse, a bayonet charge ensued which presented us with this class of wounds for the first time.

Through the courtesy of my friends, Surgeon L. Guild, Medical Director of Gen. Lee's army, and Surgeon J. T. Gilmore, Chief Surgeon of McLaws' division, and Surgeon Baruch, 3d South Carolina battalion, I examined several whose chests had been entirely transfixed by the bayonet, and who were all doing well. Their wounds healed in less than forty-eight hours; two had expectorated a little blood, but careful auscultation could detect no abnormal sounds; there was but little pain present and no cough; no hemorrhage of any account from the wound had been remarked. The men were seated up in their tents on the fourth day eating, and the cordeform and punctured wounds, indicating the heel and point of the bayonet, already healed, were well defined on the respective sides of the chest.

